# Latent Tuberculosis: A Promising New Compound to Treat Non-Replicating and Intramacrophagic Mycobacteria

**DOI:** 10.3390/biomedicines10102398

**Published:** 2022-09-26

**Authors:** Débora Leite Campos, Fernanda Manaia Demarqui, Mariana Cristina Solcia, Paula Carolina de Souza, Pedro Ivo da Silva Maia, Victor Marcelo Deflon, Fernando Rogério Pavan

**Affiliations:** 1 Tuberculosis Research Laboratory, School of Pharmaceutical Sciences, São Paulo State University—UNESP, Araraquara 14800-903, Brazil; 2 Institute of Exact, Natural Sciences and Education, Triangulo Mineiro Federal University—UFTM, Uberaba 38064-200, Brazil

**Keywords:** latent tuberculosis, *Mycobacterium tuberculosis*, treatment

## Abstract

As a biologic reservoir of *Mycobacterium tuberculosis* (*M. tb*), one-quarter of the world population is infected with the well-known latent tuberculosis (LTBI). About 5–10% of LTBI patients will progress to active disease in the first years after primary infection and, despite using the recommended treatment, 20% can still reactivate the infection. A new LTBI treatment could minimize adverse effects and antibiotic resistance that can occur when the same drug is used to treat the latent and active disease. New hydrazones were evaluated, and they showed great inhibitory activity against intramacrophagic and non-replicating *M. tb*, commonly found at this stage of infection, in addition to bactericidal and narrow-spectrum activity. When tested against eukaryotic cells, the hydrazones showed great safety at different exposure times. In vitro, these compounds performed better than isoniazid and could be considered new candidates for LTBI treatment, which may promote greater engagement in its prescription and adherence.

## 1. Introduction

Tuberculosis (TB) is an infectious disease caused by the aerobic acid-fast bacillus *Mycobacterium tuberculosis*. Despite being a treatable and preventable disease, TB still ranks among the leading causes of death in the world, with about 5.8 million new cases reported in 2020 [1]. Moreover, it is estimated that nearly a quarter of the world population is infected with the bacillus in its latent form [2,3].

Individuals with latent TB infection (LTBI) are asymptomatic and do not transmit the bacillus. Nonetheless, viable *M. tuberculosis* is present in the host, restrained within granulomas by the immune system, which leads to a 5–10% rate of people who may develop the active form of the disease [4,5]. Therefore, one step towards TB eradication is treating LTBI cases to eliminate this reservoir of bacteria [6].

Currently, a strongly indicated treatment for LTBI in countries with high and low TB incidence is isoniazid (INH) monotherapy for six months. Ongoing and recently completed clinical trials available in the database of the United States National Institute of Health (NIH) focus exclusively on alternative regimens and dosages of INH and rifampicin (RFP) or rifapentine, with combined treatment or monotherapy for reducing the time of treatment [7]. This is because the completion rate for INH monotherapy is lower than that of surrogate treatments [8,9].

Since in LBTI, *M. tuberculosis* is contained in granulomas, an ideal drug to act in this scenario should be capable of eliminating the bacillus in extra and intracellular conditions, both in the active replication or the non-replicating state. It should also act against resistant strains, have high penetration potential, show no cellular toxicity, exhibit narrow spectrum, and be effective in a short period [10].

**Figure 1:**
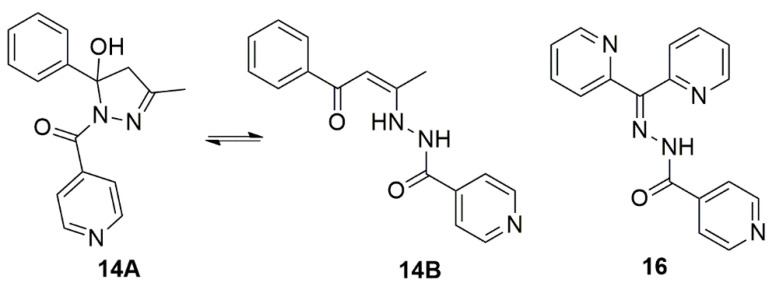
Molecular structures of the compound 14 in (A) cyclic form and (B) open chain form and compound **16**.

A common approach to developing molecules with antimicrobial potential is using known functional chemical structures as precursors of new molecules, and INH is one of the most widely employed [11,12]. Along this line, the activity against *M. tuberculosis* of twenty compounds based on the thiosemicarbazone, semicarbazone, hydrazide/hydrazone, and dithiocarbazate classes were presented by Pavan et al. [13]. Two of these hydrazones, presented in Figure 1, were selected for this study. These two compounds inhibited the growth of the bacillus and had a high selectivity index when tested in macrophage cells for 24 h.

Hydrazones exhibit many known biological activities, including antimicrobial, antidepressant, antiplatelet, anticancer, and antifungal properties, among others [14,15]. In this study, these two hydrazones were evaluated to investigate their potential as a new treatment option for LBTI. It was observed that these compounds were able to inhibit replicating, non-replicating, and intramacrophagic mycobacteria. They also exhibited bactericidal and narrow-spectrum activity with a safety profile.

## 2. Materials and Methods

### 2.1. Hydrazones and Anti-TB Drugs

Hydrazones 14 and 16 were synthesized and characterized, as previously described [13,16]. RFP and INH were purchased from Sigma-Aldrich^®^ (Burlington, MA, USA). Stock solutions were prepared in DMSO (Sigma-Aldrich^®^), at 10 mg/mL.

#### Analytical

Compound **14**: Color: colorless. Formula: C_16_H_15_N_3_O_2_∙H_2_O (MW = 299.32 g/mol). IR (ν_max_/cm^–1^): 3421 ν(O-H_water_), 3210 ν(O-H), 1632 ν(C=O), 1549 ν(C=N), 1499 ν(C=C + C–C). RMN ^1^H (400 MHz, DMSO-*d*_6,_ δ ppm): 1.99 (s, 3H, CH_3_), 2.10 (s, CH_3_, open tautomer), 3.08–3.20 (m, 2H, CH_2_), 6.02 (s, 1H, CH, open tautomer), 7.06 (s, 1H, OH), 7,29 (t, J = 7.2 Hz, 1H, Ph), 7.37 (t, J = 7.2 Hz, 2H, Ph), 7.48 (d, J = 7.2 Hz, 2H, Ph), 7.57 (d, J = 6.0 Hz, 2H, Py), 7.80 (d, open tautomer), 7.90 (d, open tautomer), 8.68 (d, J = 6.0 Hz, 2H, Py), 8.81 (d, open tautomer), 11.15 (s, 1H, NH, open tautomer), 12.28 (s, 1H, OH).

Compound **16**: Color: colorless. Formula C_17_H_13_N_5_O (MW = 303.32 g/mol). IR (ν_max_ /cm^–1^): 1696 ν(C=O), 1586 ν(C=N). ^1^H NMR (400 MHz, DMSO-*d*_6_) δ 7.54 (d, *J* = 8.0 Hz, 2H), 7.65 (t, *J* = 8.0 Hz, 1H), 7.84–7.79 (m, 2H), 7.95 (d, *J* = 7.3 Hz, 1H), 8.09–7.97 (m, 2H), 8.58–8.68 (m, 1H), 8.88–8.82 (m, 2H), 9.00–8.95 (m, 1H), 15.24 (s, 1H, NH).

### 2.2. Bacterial Culture

*Mycobacterium tuberculosis* H_37_Rv (ATCC 27294) and *Mycobacterium smegmatis* mc^2^155 (ATCC 700084) were cultured in Middlebrook 7H9 broth supplemented with 10% OADC (oleic acid, albumin, dextrose, and catalase) for ten and two days under shaking conditions (120 rpm), at 37 °C, respectively. For spectrum activity, the *Salmonella Typhimurium* (ATCC 14028), *Pseudomonas aeruginosa* (ATCC 13388), *Escherichia coli* (ATCC 11775), and *Staphylococcus aureus* (ATCC 6538) were cultured in Luria–Bertani broth for 20 h at 200 rpm and 37 °C.

### 2.3. Minimal Inhibitory Concentration (MIC_90_) Evaluation against Replicating M. tuberculosis and M. smegmatis

MIC_90_ was determined according to Palomino et al. [17]. The bacteria were cultured until the optical density of McFarland no. 1 standard and diluted to 10 × 10^5^ CFU/mL (colony forming unit). In a 96-well plate, 100 μL of the compounds were plated in a range of 0.09 to 25 μg/mL, and 100 μL of mycobacterial solution was added. The plate was incubated at 37 °C and 5% CO_2_ atmosphere for seven and two days for *M. tuberculosis* and *M. smegmatis*, respectively. Resazurin (30 μL–0.1 mg/mL) was added and, after 24 h, the fluorescence was read at 530/590 nm. The MIC_90_ was determined as the mean of three biological replicates.

### 2.4. Minimal Inhibitory Concentration Evaluation against Non-Replicating M. tuberculosis

The LORA method was used to determine the MIC_90_ of compounds against latent/non-replicating (NRP) mycobacteria. According to Wayne and Sohaskey (2001) [18], under a low-oxygen atmosphere, NRP mycobacteria, containing the plasmid pFCA-ABCDE that encodes a luminescent protein in aerated environments, were plated in a 96-well plate with 100 mL of compounds in a range of 0.39 to 100 μg/mL. The plates were incubated under the conditions described by Solcia et al. (2021) [19], and the MIC was defined as the lowest concentration of the compound capable of inhibiting the multiplication of 90% of latent *M. tuberculosis* cells [20].

### 2.5. Cytotoxicity against Eukaryotic Cells

For the cytotoxicity assays, MRC-5 (ATCC CCL-171) and J774A.1 (ATCC TIB-67) cells were thawed and cultured in DMEM (Vitrocell^®^, Waldkirch, Germany) and RPMI (Vitrocell^®^, Waldkirch, Germany) medium, respectively, supplemented with 10% of fetal bovine serum (FBS), gentamicin sulfate (50 mg/L), and amphotericin B (2 mg/L). A volume of 2.5 × 10^5^ cells/mL of MRC-5 and 1.0 × 10^6^ cells/mL of J774A.1 were seeded in a 96-well microplate, with a final volume of 100 µL and incubated for 24 h at 37 °C with 5 % CO_2_ to allow cell adhesion. Compounds were added in a dilution range of 0.39–100 μg/mL with 24 h, 48 h, and 72 h incubations at 35 °C and 5% CO_2_. Then, 50 μL of 0.01 % resazurin solution (solubilized in sterile distilled water) was added and, after 3 h, the fluorescence reading was performed using the Synergy H1 reader (Biotek^®^, Santa Clara, CA, USA). The IC_50_ value was defined as the lowest concentration of hydrazones at which 50% of the cells remained viable [18].

### 2.6. Determination of the Selectivity Index

By dividing the IC_50_ values by the MIC_90_, it is possible to identify the safest compounds when the result is greater than 10 [21].

### 2.7. Intramacrophagic Activity

Intramacrophagic activity was determined by in vitro infection of macrophages by *M. tuberculosis* H_37_Rv. The J774A.1 macrophage cells were cultured in RPMI medium supplemented with 10% FBS, and 5 × 10^5^ cell/mL was seeded into wells of a 24-well plate and incubated at 35 °C and 5% CO_2_ atmosphere for 24 h for cell confluency. Phagocytosis was performed with incubation of 10^5^ CFU/mL of H37Rv inoculum for 2 h. Amikacin solution at 200 μg/mL was used to sterilize the extracellular medium. Hydrazone solutions, diluted in RPMI medium at values equal to or four times higher or lower than its MIC_90_, were added and the RFP was used in the same conditions as control. After 72 h, the cells were lysed with 1 mL of 0.1% Triton, and the solution was serially diluted and plated in Middlebook 7H10 medium. After 30-day incubation at 37 °C and 5% CO_2_, CFU were counted. The result is shown as the average of two independent studies of intracellular bacteria, compared to the growth of the untreated wells at 72 h [22]. One-way analysis of variance (ANOVA) followed by Dunnett’s *post-hoc* test was used to calculate statistical differences from non-treated group and *p* values of less than <0.05 were considered statistically significant.

### 2.8. Time-Kill Curve

Hydrazones were prepared in DMSO at 10 mg/mL. This solution was then diluted in Middlebrook 7H9 medium (Difco^®^, Franklin Lakes, NJ, USA) supplemented with OADC, so that its final concentration in contact with the mycobacteria was equal to or twice greater than the MIC_90_ value previously established by the REMA method. The initial concentration of *M. tuberculosis* in solution was 5 × 10^5^ CFU/mL. As a control of the experiment, a culture medium containing only mycobacteria at the same concentration was used, in addition to RFP and INH at the concentrations of MIC and 2× MIC. The samples were incubated in an orbital shaker at 37 °C and 121 rpm for 15 days and aliquots were taken, serially diluted, and plated every 48 h. Solid medium plates were incubated at 37 °C in a 5% CO_2_ atmosphere for 20–30 days to count CFU/mL. The experiment was carried out in three independent assays, and the results are expressed as the arithmetic mean and standard error of the number of CFU/mL. With the values obtained, it was possible to draw a curve representing the number of viable bacteria in decimal logarithm (ordinate) as a function of exposure time in days (abscissa) [23].

### 2.9. Evaluation of Activity Spectrum

*E. coli*, *S. typhimurium*, *P. aeruginosa*, and *S. aureus* were cultured as described below and used to evaluate the activity spectrum. The microdilution assays were performed following the protocol established by the CLSI manual “Methods for Dilution Antimicrobial Susceptibility Tests for Bacteria That Grow Aerobically” (2018) [24], with modifications: the bacterial strains were cultured in liquid LB medium for 20 h in an orbital shaker at 200 rpm and 37 °C. They were then diluted to 5 × 10^5^ CFU/mL, and 100 µL of inoculum was added to another 100 µL of compounds at concentrations of 0.39–100 µg/mL. After 24 h, the absorbance reading was performed at 600 nm in a Synergy H1 plate reader (Biotek^®^, Santa Clara, CA, United States). The result is shown as the average of three independent assays [19].

## 3. Results

### 3.1. Minimum Inhibitory Concentration (MIC90) Values against M. tuberculosis in an Active and Non-Replicating Metabolic State

The results of mycobacteria inhibition by hydrazones are presented in Table 1. All hydrazones were able to inhibit non-replicating mycobacteria, with MIC_90_ values of 6.31 and 7.76 μM for hydrazone 14 and 16, respectively. These results support the hypothesis that these compounds are new promising drugs for LTBI treatment, mainly when these data are compared with the MIC value obtained in this experiment for INH, with a MIC value higher than 300 μM. In addition, the MIC_90_ values against replicating bacteria are also promising, with MIC values of 1.23 and 6.01 μM for hydrazone 14 and 16, respectively.

### 3.2. Cytotoxicity Index (IC_50_) and Selectivity Index (SI) Results against Macrophage Cells and Lung Fibroblasts at 24, 48, and 72 h

*In vitro* cell viability was performed with hydrazones against MRC-5 and J774A.1 cells, and the results obtained using resazurin are summarized in Table 2. The objective of this test was to evaluate the performance of hydrazones at different exposure times in concentrations of up to 100 μg/mL.

MRC-cell (normal human fetal lung fibroblasts) was selected because it is widely used for the phenotypic screening of compounds and J774A.1, a mice macrophage original cell line. This cell is usually used to determine the inhibition of intracellular pathogens and, because of the incubation time of the intramacrophagic experiment, it was tested for up to 72 h [25,26].

After 24 h of exposure, both hydrazones showed IC_50_ values higher than 100 μg/mL against MRC-5 and J774A.1. Hydrazone 14 demonstrated no detectable cytotoxicity when tested against MRC-5 at all exposure times. Hydrazone 16 showed an increase in toxicity proportional to the increase in exposure time with an IC_50_ value of 72 μg/mL after 72 h. Despite this, this result is still optimal when the SI is calculated, resulting in a safety profile with a value greater than 39. Table 2 also presents the SI obtained for hydrazones and shows that hydrazone 14 was safer, with SI values higher than 200 in all evaluated cells and times.

### 3.3. Intramacrophagic Activity

**Figure 2:**
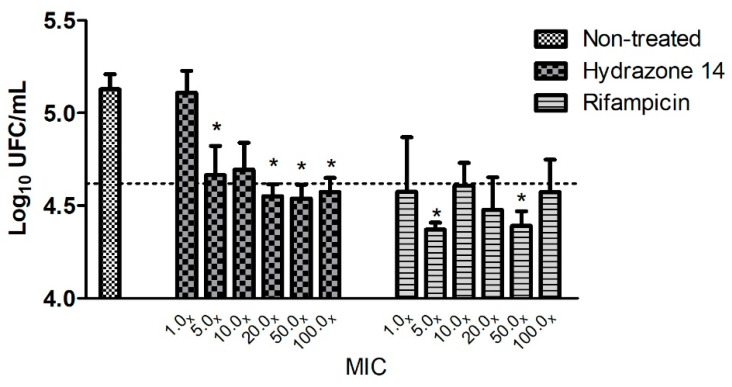
Intramacrophagic activity of hydrazone 14 and rifampicin at different MICs after 3 days of exposure. The results are presented as the mean and standard deviation of three independent experiments. The line represents an inhibition of 1 log_10_ CFU/mL compared to non-treated control. * *p* < 0.05, statistically significant differences found compared to the non-treated group.

The intracellular antimycobacterial activity of hydrazone 14 was investigated using the adherent macrophage J774A.1 infected with a 2 MOI of *M. tuberculosis*. The inhibition of mycobacteria is demonstrated as a log reduction value in comparison with the untreated sample in Figure 2.

Figure 2 shows that, for hydrazone 14, at a concentration of 5× MIC (6.15 μM), inhibition of 0.5 log_10_ CFU/mL is detectable. Increases in concentration above this value do not promote a substantial increase in intramacrophagic inhibition. Despite this, this similarity to RFP is a considerably good result.

### 3.4. Time-Kill Curve

**Figure 3:**
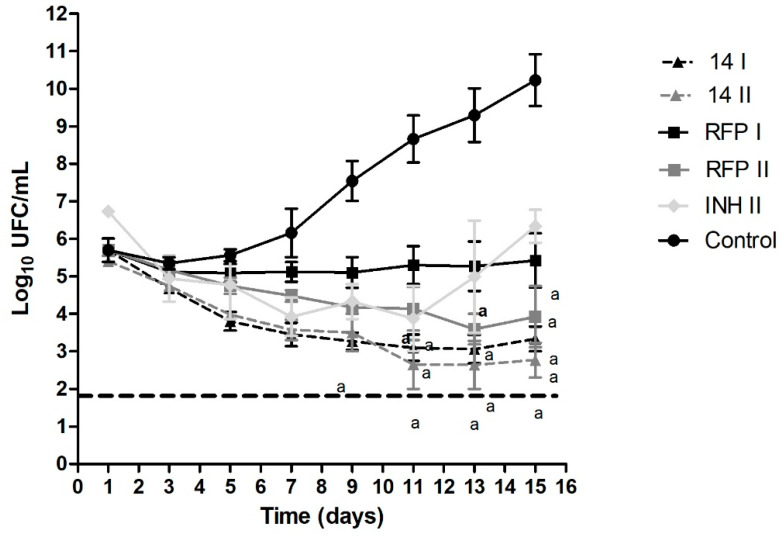
Time-kill curve of hydrazone 14 at 1× (14 I) and 2× (14 II) MIC and controls: rifampicin (RFP) 1× and 2× MIC, isoniazid (INH) 2× MIC and negative control (control). Dotted line represents a reduction of 3 log_10_ CFU/mL. Statistical analysis was performed using Prism 5.0. The data were analyzed using one-way analysis of variance, followed by Newman–Keels post-hoc test. The results are presented as the mean ± standard deviation from three independent experiments; a: *p* < 0.05 compared to control group.

**Figure 4:**
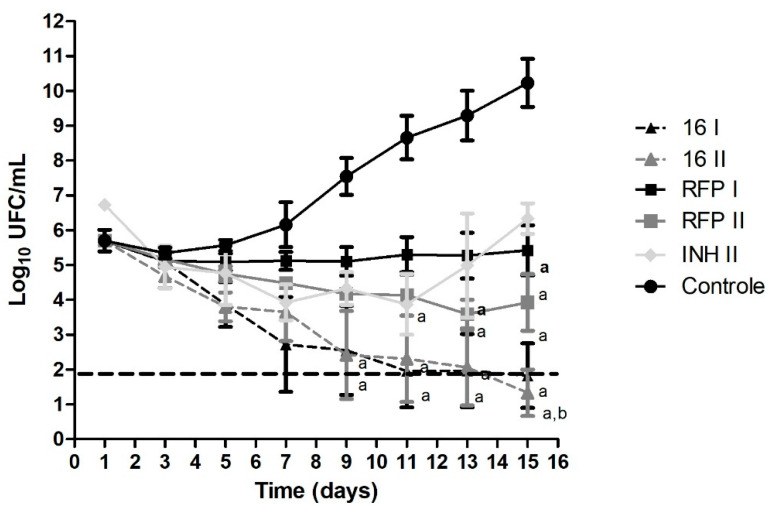
Time-kill curve of hydrazone 16 at 1× (16 I) and 2× (16 II) MIC and controls: rifampicin (RFP) 1× and 2× MIC, isoniazid (INH) 2× MIC, and negative control (control). Dotted line represents reduction of 3 log_10_ CFU/mL. Statistical analysis was performed using Prism 5.0. The data were analyzed using one-way analysis of variance, followed by Newman–Keels post-hoc test. The results are presented as the mean ± standard deviation from three independent experiments; a: *p* < 0.05 compared to control group. b: *p* < 0.05 compared to INH.

The results obtained from the time–kill assay are presented in Figure 3 and Figure 4, for hydrazones 14 and 16, respectively. We determined the death profile of the compounds by observing the bacterial growth curve in treated and untreated conditions (control). Both hydrazones were tested at their MIC and 2× MIC values so that any change in the death profile caused by the hydrazone at a higher concentration could be evaluated.

Both compounds were bactericidal in the conditions tested. The bactericidal activity is characterized by a 99.9% decrease in the bacterial inoculum (three units at logarithmic scale) [12,23,27]. RFP and INH were used as controls at the same concentrations, and both molecules showed a bacteriostatic effect in vitro, as expected at these concentrations.

### 3.5. Spectrum Activity

The activity spectrum represents the compound’s ability to inhibit the growth of different bacterium species. The results shown in Table 3 were obtained as the average of three independent trials. The hydrazones did not inhibit the growth of the gram-negative or gram-positive bacteria tested, exhibiting a narrow-spectrum activity, which is a good characteristic for a new compound for TB treatment. When tested against *M. smegmatis*, hydrazone 14 was unable to inhibit the mycobacteria growth, demonstrating that the inhibitory effect is specific against *M. tuberculosis*.

## 4. Discussion

The investigation and treatment of LTBI in at-risk populations is a known form of prevention that decreases the number of active TB cases in a given region. The diagnosis is based on the immune responses to *M. tuberculosis* antigens even if there are no obvious symptoms of infection [28,29].

It is estimated that one-quarter of the world population is infected with *M. tuberculosis*, behaving as a biological reservoir of the bacilli [28,30]. Therefore, the treatment of this population has become an important factor in the control and eradication of TB, especially because 5–10% of infected individuals may progress to active disease [30].

There are four recommended treatments against LTBI, as shown in Table 4 below. The main scheme is INH monotherapy. This treatment scheme was first implemented in the 1960s by recommendation of the American Thoracic Society [31,32]. It is known that *M. tuberculosis* can be non-replicating after initial infection. This “dormant” state is promoted by its immune-evading capability, which allows persistent infection without fast multiplication and, because of this non-replicating state, some standard drugs used in TB treatment lose their effectiveness due to their mechanisms of action [33].

For example, in the case of INH, which inhibits the InhA protein related to mycolic acid synthesis, in a non-replicating situation in which a large production of these membrane components is unnecessary, the inhibition of INH is not effective, as reflected in the results of the LORA assay, where INH presented non-inhibition against non-replicating bacteria [34].

Thus, considering LTBI a major non-replicating condition of bacteria, the hydrazones studied in this project have higher effectiveness in inhibiting *M. tuberculosis* growth than INH, at least *in vitro*. Treatment with INH in LTBI cases showed good activity [32] even though it was effective only against replicating bacteria. However, a drug with inhibitory effects against both bacterial metabolisms, such as hydrazones, could be even better. The MIC values observed for these compounds were much lower than those of INH when tested against non-replicating bacteria in the LORA experiment. This preliminary result opens the possibility to investigate hydrazones as new promising candidates for the LTBI treatment.

When the MIC against replicating mycobacteria is analyzed, it is important to consider that even though the values are higher in vitro than those of the standard drugs for TB treatment, we are considering these hydrazones for exclusive use as a new LTBI treatment. Treating patients with specific compounds during a latent infection is beneficial, mainly in the case of TB activation after LTBI treatment, which occurs in 10% of the cases when INH monotherapy is used [28]. These benefits are (1) reduced risk of promoting resistance to the current treatment [29], (2) no side effects after long use of the same antibiotic [35], and (3) shorter treatment schemes using alternative drugs. In this regard, a very low MIC value against replicating bacteria turns out to be the least important factor and the use of hydrazones with a good MIC value and high safety profile remains an option as a drug treatment in LTBI cases.

Hydrazones yielded another important result when tested against eukaryotic cells at different exposure times. Against murine macrophages, no statistical differences were observed between hydrazones and INH at all evaluated exposure times, demonstrating that the safety of hydrazones and INH have similar safety profiles.

When tested against human fibroblasts, hydrazone 14 was always similar to INH, while hydrazone 16 only showed a statistical difference when compared to INH after 72 h of exposure. Nevertheless, the SI exhibited by hydrazone 16 at all exposure times was higher than 10, which points to the non-toxic profile of this compound. The capacity of compounds to inhibit intracellular mycobacteria is very important due to granuloma formation, which is an essential step in infection control [36]. The granuloma found in LTBI is formed by a combination of heterogenic immune cells, including macrophages, T and B cells, neutrophils, fibroblasts, and dendritic cells at different concentrations. In this environment, bacteria are found inside many of these cell types. When phagocytized, using its mycobacterial machinery to block the lysis inside macrophage phagosome, the bacillus can replicate and spread uncontrollably. In this way, intramacrophagic inhibition can improve the elimination effect of antibiotics [36,37].

Hydrazone 14 was able to inhibit *M. tuberculosis* in both conditions and showed an inhibitory intracellular effect very similar to that of RFP in the same conditions. A decrease was observed in the growth of 0.5 log_10_ CFU/mL, which represents 45% of inhibition in 3 days of exposure as observed with RFP. No statistical differences were found between hydrazone 14 and RFP at concentrations of 2x to 100x MIC. The inhibition of only 0.09 log_10_ CFU/mL at 1x MIC was observed for hydrazone 16 (data not shown).

Then, in an LTBI treatment, the use of compounds that act against non-replicating and intramacrophagic bacteria is the best way to control the disease, providing a shorter treatment scheme that can promote better patient adherence and fewer adverse effects [28]. Additionally, an antibiotic with a narrow-spectrum activity can offer greater safety to an LTBI treatment.

Broad spectrum antibiotics have a determinant role in the treatment of some diseases. However, when it comes to the prolonged treatment of specific bacteria, such as in the case of TB, this broad spectrum may lead to undesirable effects, due to the development of microbial resistance and permanent damage to the microbiome [38].

The hydrazones were inactive when tested against gram-positive and gram-negative bacteria, and they could not inhibit the growth of *M. smegmatis*. Therefore, they had a narrow-spectrum activity, being highly selective against *M. tuberculosis*.

In the current LTBI treatment, INH and RFP have different spectrum profiles. INH is a narrow-spectrum antibiotic, while RFP has a broad-spectrum activity; the latter inhibits the growth of most gram-positive bacteria and numerous gram-negative microorganisms, which may perturb patients’ microbiome, causing gastrointestinal symptoms [28,39].

In addition to the narrow spectrum, the killing rate of a compound is also an important characteristic to be observed. Ideally, an anti-TB drug should present a high killing rate and be active against mycobacteria in a low-growth state [23].

With regard to its killing efficiency, a drug can be classified as bactericidal, when it can eliminate all bacteria, or bacteriostatic, when it inhibits bacteria growth but does not kill them. Based on our experiments, both hydrazones showed a bactericidal profile while RFP and INH were bacteriostatic in the same conditions. Therefore, hydrazones could be considered core drugs for LTBI treatment, i.e., drugs that promote a cure with no relapses.

The mechanism of action of hydrazones was also investigated through the analyses of *M. tuberculosis* plasmatic membrane. Phenotypic experiments were performed to observe the inhibition of mycolic acid synthesis and efflux pumps [19]. These data are not shown because the hydrazones were not able to inhibit any of these membrane structures. Further experiments need to be performed to identify the hydrazones’ mechanism of action.

Chemically, both compounds are isoniazid derivatives, however, there are significant structural modifications between them, which might point out an explanation for their different biological activities. For example, the modifications of the groups attached to the hydrazone moiety influence their lipophilicity, promoting an expected increase in Log P for both compounds, when compared to isoniazid, which is probably also related to their distinct antibacterial effects.

As compound **14** can be obtained in the hydrated cyclic hydrazone form (**14A**∙H_2_O) as a colorless solid [40,41] or in the open hydrazide form (**14B**) as a yellow crystalline solid whose crystal structure was previously reported by our group [13], it is important to explain that, in the present work, a colorless solid was obtained for compound **14** and the ^1^H NMR spectrum from DMSO-*d*_6_ solution of this compound shows that the cyclic form is predominant in the solution. On the other hand, the ^1^H NMR spectrum of the compound **16** suggests the presence of only one specimen in solution.

## 5. Conclusions

Given the in vitro potency of hydrazones presented here and considering that some in vivo studies are necessary, we could consider these compounds as new promising candidates by its capacity in inhibiting non-replicating and intramacrophagic mycobacteria, their bactericidal and narrow spectrum activity, and their safety against eukaryotic cells at different exposure times. The standard scheme of INH monotherapy, although functional, causes many adverse effects and could promote resistance if infection reactivates. An exclusive treatment for LTBI must be investigated in order to provide practitioners with a prescription option that should result in greater treatment adherence and TB eradication.

## Figures and Tables

**Table 1:** Minimum inhibitory concentration (MIC_90_) of hydrazones and control drugs against *Mycobacterium tuberculosis* in a replicating and non-replicating metabolic state.

Compounds	MIC_90_^1^ Replicating		MIC_90_ Non-Replicating	
	μM	μg/mL	μM	μg/mL
Hydrazone 14	1.23 ± 0.53	0.35 ± 0.15	6.31 ± 5.72	1.77 ± 1.61
Hydrazone 16	6.01 ± 0.22	1.82 ± 0.68	7.76 ± 5.70	2.35 ± 1.73
Rifampicin	0.05 ± 0.001	0.04 ± 0.01	<0.36	<0.3
Isoniazid	0.44 ± 0.29	0.06 ± 0.04	>328.13	>45.0

^1^ MIC: minimum inhibitory concentration.

**Table 2:** Inhibitory concentration (IC_50_) and selectivity index (SI) of hydrazones 14, 16, and isoniazid against macrophages (J774A.1) and lung fibroblasts (MRC-5) at 24, 48, and 72 h.

		IC_50_ (μg/mL)			IS		
Cells	Time	H14	H16	INH	H14	H16	INH
J774A.1	24 h	>100	>100	>100	>289.02	>54.85	>1666.66
	48 h	83.34 ± 28.85	95.60 ± 7.61	>100	240.88	52.44	>1666.66
	72 h	81.81 ± 31.50	72.29 ± 47.99	>100	236.46	39.65	>1666.66
MRC-5	24 h	>100	>100	>100	>289.02	>54.85	>1666.66
	48 h	>100	96.23 ± 6.53	>100	>289.02	52.79	>1666.66
	72 h	>100	67.43 ± 28.66 *	>100	>289.02	36.99	>1666.66

H14 = hydrazone 14; H16 = hydrazone 16. The IC_50_ data were analyzed using two-way analysis of variance, followed by Bonferroni post-hoc test. * *p* < 0.01 compared to INH.

**Table 3:** Spectrum activity of hydrazones against gram-positive and negative bacteria and *Mycobacterium smegmatis*. Results as a medium ± standard deviation in μg/mL.

Molecules	*S. typhimurium*	*P. aeruginosa*	*S. aureus*	*E. coli*	*M. smegmatis*
14	>100	>100	>100	>100	>25.0
16	>100	>100	>100	>100	n.d.
Gentamicin	1.16 ± 0.38	2.02 ± 0.66	3.97 ± 2.35	5.07 ± 0.14	n.d.
Rifampicin	n.d. *	n.d.	n.d.	n.d.	2.4 ± 1.5

* n.d. = not determined.

**Table 4:** Recommended treatments against latent tuberculosis infection (LTBI) and its efficacy in preventing progression to active tuberculosis infection.

Scheme Treatment	Treatment Period	Efficacy
Isoniazid monotherapy	6–9 months	90%
Rifampicin monotherapy	4 months	~90%
Isoniazid and Rifampicin	3–4 months	n.p.
Isoniazid and Rifapentine	3 months	n.p.

Reference: Huaman et al. (2019) [28]; n.p. = not presented.
